# Feasibility Study of Renal Negative Pressure Treatment After Cardiac Surgery With Cardiopulmonary Bypass

**DOI:** 10.1016/j.atssr.2026.01.017

**Published:** 2026-02-26

**Authors:** Evelio Rodriguez, Milovan Petrovic, Krzysztof Milewski, Slobodan Micovic, Andrej Preveden, Krzysztof Sanetra, Dragana Kosevic, Robert N. Sladen

**Affiliations:** 1 Division of Cardiac Surgery, University of Tennessee, Ascension Saint Thomas Heart West, Nashville, Tennessee; 2 Faculty of Medicine, University of Novi Sad and Institute of Cardiovascular Diseases of Vojvodina, Sremska Kamenica, Serbia; 3 Faculty of Medicine, Academy of Silesia Katowice and Center for Cardiology and Cardiac Surgery, American Heart of Poland, Bielsko-Biala, Poland; 4 Dedinje Cardiovascular Institute, University of Belgrade, Belgrade, Serbia; 5 Division of Critical Care Medicine, Department of Anesthesiology, Columbia University Irving Medical Center, New York, New York

## Abstract

**Background:**

We conducted a technical feasibility and safety study of intraureteric renal negative pressure treatment (rNPT) in patients with chronic kidney disease undergoing elective cardiac surgery with cardiopulmonary bypass

**Methods:**

After written informed consent, 10 patients with preoperative estimated glomerular filtration rate of 15 to 59 mL/min per 1.73 m^2^ were enrolled at 3 sites. At the end of surgery, a urologist advanced a specialized ureteral catheter into each renal pelvis under radiographic guidance. Intra.renal negative pressure of −15 mm Hg was initiated postoperatively and continued for 24 hours. Patients were followed up on postoperative days 14 and 28.

**Results:**

Three patients had major protocol deviations with primary catheter misplacement; acute kidney injury developed in 1 of these. Bilateral renal pelvis catheters were deployed per protocol in 7 patients. All patients had microhematuria; 2 had transient hematuria. In the per-protocol group, acute kidney injury developed in 1 patient with stage 4 chronic kidney disease. No patient suffered any other major adverse event. Serum creatinine concentration remained normal in 6 patients at 72 hours; and in 5 patients, estimated glomerular filtration rate had improved from baseline at 28 days.

**Conclusions:**

rNPT is feasible after cardiac surgery. If catheter placement protocol is strictly followed, it is associated with minimal patient risk and potential benefit. A controlled study (NCT07017933) is in progress to evaluate rNPT started before cardiopulmonary bypass and continued postoperatively.

**ClinicalTrials.gov identifier:**

NCT05990660

**In Short**

▪ Renal negative pressure treatment (rNPT) can enhance glomerular filtration rate by decreasing renal venous pressure and glomerular afterload.
▪ Ten patients with preexisting chronic kidney disease undergoing cardiac surgery with cardiopulmonary bypass were studied. In the 7 in whom there was strict adherence to catheter placement protocol, application of rNPT was feasible and safe.
▪ Further studies are required to evaluate the renoprotective effect of rNPT applied throughout cardiac surgery with cardiopulmonary bypass and to identify the population most likely to benefit.

Renal injury complicating cardiac surgery with cardiopulmonary bypass (CPB) is associated with increased adverse events, intensive care unit (ICU) and hospital length of stay, and 30-day mortality.^1^ Its potential is increased by chronic kidney disease (CKD).

Glomerular filtration rate (GFR) is impaired by inadequate arterial perfusion (decreased renal preload) or venous congestion (increased renal afterload).^2^ Therapies for congestive heart failure (CHF) and cardiorenal syndromes include aggressive diuresis and devices to reduce venous congestion.^3^^,^^4^

Renal negative pressure treatment (rNPT) involves placement of specialized ureteral catheters into each renal pelvis. An attached vacuum pump generates continuous mild negative pressure with the goal of decreasing excessive renal afterload and maintaining GFR under adverse conditions.

In a porcine model of CHF, rNPT enhanced GFR, diuresis, natriuresis, and fluid balance.^5^ Application of −15 mm Hg rNPT yielded similar benefits in patients with decompensated CHF.^6^ We designed a technical feasibility and safety study in patients with CKD to evaluate rNPT after cardiac surgery with CPB.

## Patients and Methods

This prospective open-label investigational device study was conducted at 3 sites in Serbia and Poland: Institute of Cardiovascular Diseases of Vojvodina, Sremska Kamenica; Dedinje Cardiovascular Institute, Belgrade; and Cardiac Surgery Center, American Heart of Poland, Bielsko-Biala. The protocol was approved by the Medicines and Medical Devices Agency of Serbia and the Office for Registration of Medicinal Products, Medical Devices, and Biocidal Products in Poland. Written informed consent was obtained from all patients.

Candidates for the study were patients aged 22 to 85 years undergoing cardiac surgery with CPB with a one-time baseline estimated GFR (eGFR) of 15 to 59 mL/min per 1.73 m^2^ (CKD stage 4, 3b, or 3a).

**Figure 1:**
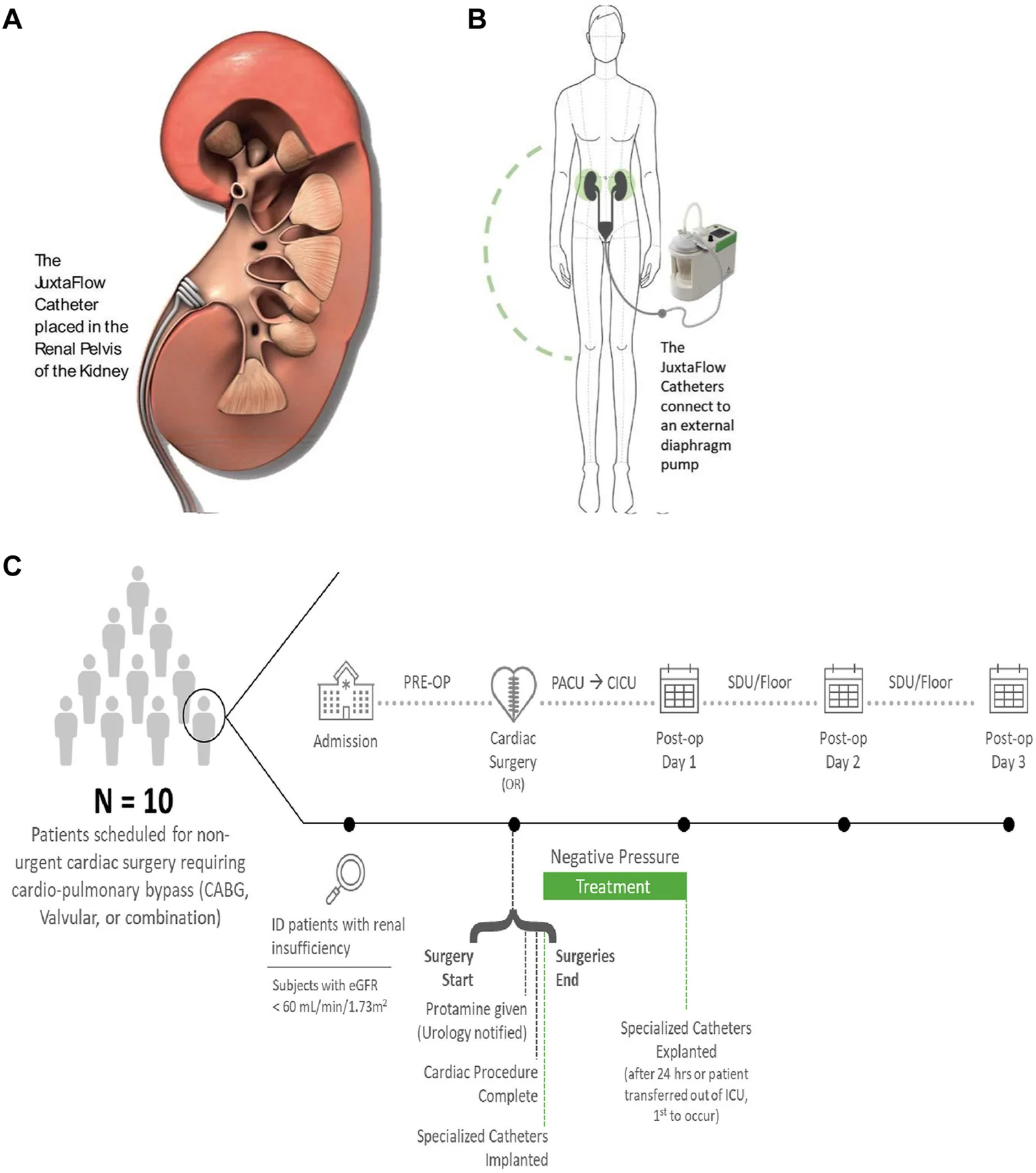
Renal assist device and study design. (A) Each JuxtaFlow catheter is advanced endoscopically up the ureter over a guidewire under fluoroscopic guidance. A specialized memory coil unfolds when the tip enters the renal pelvis to hold it gently in place. (B) The 2 JuxtaFlow catheters are attached to a diaphragm pump that generates continuous negative pressure treatment of −15 mm Hg. Illustrations courtesy of 3ive Labs, LLC. (C) Study design. (CABG, coronary artery bypass grafting; CICU, cardiac intensive care unit; eGFR, estimated glomerular filtration rate; ICU, intensive care unit; ID, identify; OR, operating room; PACU, postoperative anesthesia care unit; Post-op, postoperative; PRE-OP, preoperative; SDU, stepdown unit.)

After surgical closure and protamine administration in the operating room and under anesthesia, specialized bilateral ureteral catheters (JuxtaFlow; 3ive Labs) were inserted into the urethra alongside the existing urinary catheter by a urologist. After advancement under radiographic guidance into each renal pelvis, a memory polymer allowed the tip to expand into a cylindrical helix. The catheters were connected to a bedside vacuum pump generating −15 mm Hg negative pressure (Figures 1A, 1B). On admission to the ICU, rNPT was applied in the supine position for up to 24 hours postoperatively. Blood and urine samples were collected to assess kidney function for 72 hours and at postoperative days (PODs) 14 and 28 (Figure 1C).

The primary study end point was the incidence and severity of postoperative adverse events, including catheter malposition, hematuria, hospital readmission, and all-cause 30-day mortality. Secondary end points included 6-hour urinary creatinine clearance (CrCl) during rNPT, daily serum creatinine (SCr) on PODs 1 to 3, and calculation of eGFR at PODs 14 and 28. The incidence and severity of acute kidney injury (AKI) were defined by Kidney Disease Outcomes Quality Initiative SCr criteria within 72 hours postoperatively.

Statistical analysis included descriptions of the type, frequency, and severity of postoperative adverse events and their possible relationship to rNPT.

## Results

**Supplemental Figure 1:**
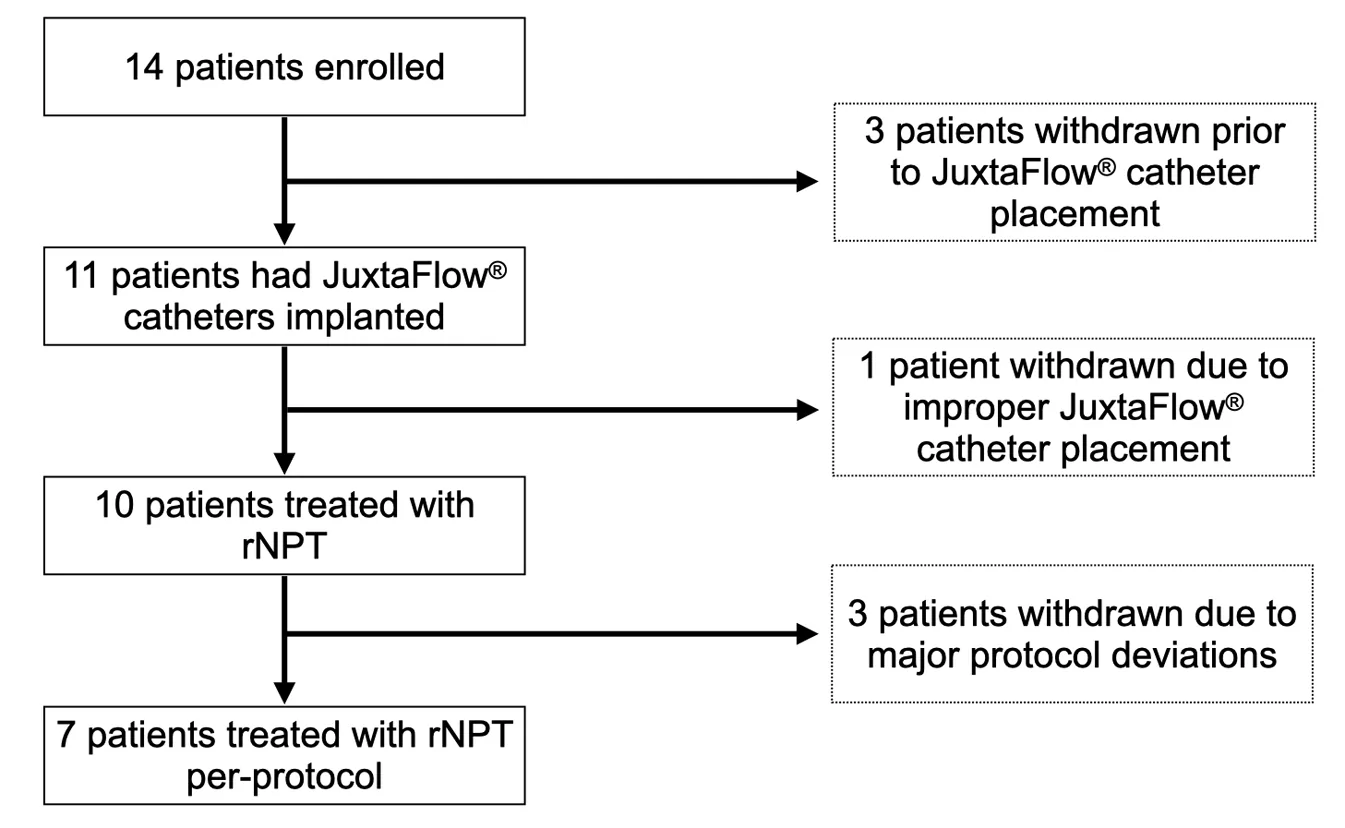
Patient population. Two patients were withdrawn because surgery was cancelled for other reasons, and one because eGFR exceeded the inclusion criteria. Eleven patients had catheters implanted at the end of the surgical procedure. One patient was withdrawn from the study because fluoroscopic guidance was not used to confirm JuxtaFlow® catheter placement and in the ICU imaging revealed that both were in the bladder. No adverse events were noted in this patient on follow up through hospital discharge.

Fourteen patients were enrolled between September 27, 2023, and January 12, 2024 (Supplemental Figure 1). Four patients were withdrawn before rNPT. The 10 remaining patients were of White race with equal sex distribution; 6 underwent coronary artery bypass grafting, 3 valve plus coronary artery bypass grafting, and 1 valve surgery. Three patients (labeled 1-3) underwent rNPT but had major protocol deviations in catheter placement, leaving 7 patients (labeled A-G) to be evaluated as the per-protocol rNPT population.

### Protocol Deviations

In 2 patients, fluoroscopy was not used until the patients were admitted to the ICU, where it was found that they had unilateral catheter placement only. In the third patient, the right JuxtaFlow catheter lodged in midureter with the helix distorted but was not repositioned. During rNPT, the patient had oliguria that rapidly resolved when the catheters were removed, consistent with obstructive uropathy. By POD 2, the SCr concentration had increased from 120 to 196 μmol/L (stage 1 AKI); elevated C-reactive protein suggested ureteric inflammation. At POD 14, SCr concentration had improved to 169 μmol/L; but at POD 28, it was elevated to 208 μmol/L.

### Per-protocol Population

Patient demographics, intraoperative events, and length of stay data are listed in Table 1. At baseline, 5 patients had stage 3a, 1 stage 3b, and 1 stage 4 CKD (Supplemental Table 2). All 7 patients underwent rNPT for a full 24 hours.

**Table 1:** Per-Protocol Patient Demographics, Perioperative Events, and Length of Stay (n = 7)

Patient demographics	Median (IQR)
Age, y	70 (68-74)
Height, cm	166 (156-167)
Weight, kg	80 (67-84)
Ejection fraction, %	49 (40-55)
CVP, mm Hg	6 (5-10)
Serum creatinine, μmol/L	105 (102-150)
eGFR, mL/min per 1.73 m^2^	52 (39-57)
Perioperative events	Mean (±SD)
Duration of CPB, min	60.6 (33.2)
Duration of surgery, min	160.7 (40.3)
Catheterization time,^a^ min	29.6 (14.2)
End of surgery to start of rNPT, min	93.3 (25.3)
Duration of rNPT, min	1444.4 (5.7)
Length of stay	Median (IQR)
Hospital,^b^ d	10 (8-11)
ICU, d	3 (3-6)
Postoperative,^c^ d	8 (7-9)

CPB, cardiopulmonary bypass; CVP, central venous pressure; eGFR, estimated glomerular filtration rate; ICU, intensive care unit; IQR, interquartile range; rNPT, renal negative pressure therapy.

a Time to place JuxtaFlow catheters.

b Hospital admission to hospital discharge.

c Day of surgery to hospital discharge.

Urine color revealed 6 episodes of transient hematuria in 2 patients (Supplemental Table 1), and urinary sediment revealed microhematuria in all patients. No patient had a urinary tract infection. Elevated C-reactive protein developed in 1 patient; oral herpes was noted at 28-day follow-up. AKI developed in 1 patient (patient D). No other postoperative adverse events were considered to be related to rNPT. No patient required return to the operating room, renal replacement therapy, or hospital readmission, and there were no cerebrovascular accidents or deaths within 30 days of surgery. Adverse events for all 10 patients are summarized in Table 2.

**Table 2:** Adverse Events Related to Study Procedures

Adverse Event	Per-Protocol Population		Protocol Deviation	
	Episodes	Patients (n = 7)	Episodes	Patients (n = 3)
Total adverse events	11	7	5	3
Non-urinary infections	1	1	1	1
Increased CRP	2	2	0	0
Hematuria	6	2	0	0
Microhematuria	7	7	3	3
AKI	1	1	1	1

AKI, acute kidney injury; CRP, C-reactive protein.

**Figure 2:**
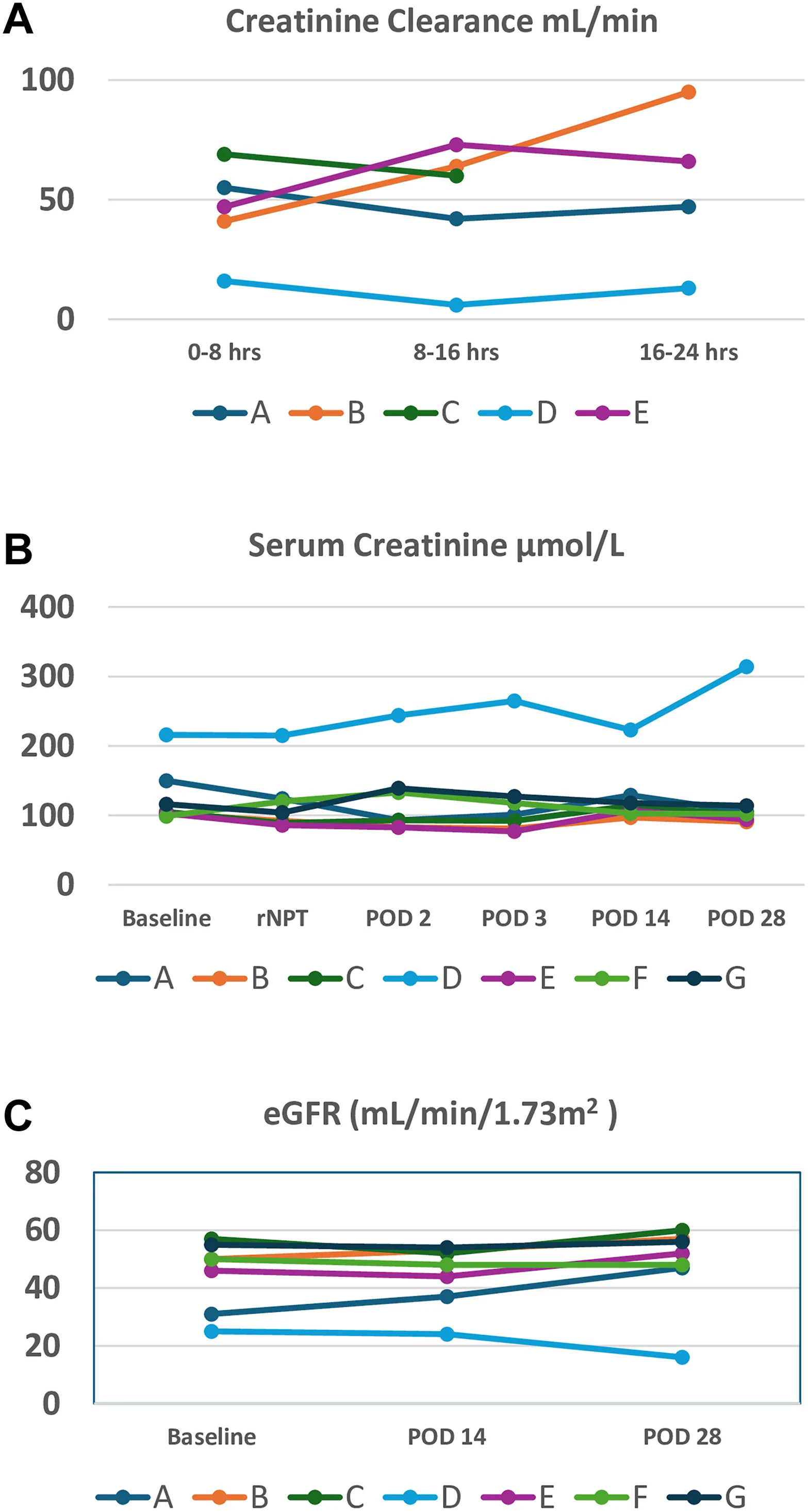
(A) Measured creatinine clearance during consecutive 8-hour phases of renal negative pressure treatment (0-8, 8-16, 16-24 hours) in 5 patients (A-E) in the per-protocol population. (B) Serum creatinine concentration in the per-protocol population (patients A-G). (C) Estimated glomerular filtration rate (eGFR) in the per-protocol population (patients A-G). (POD, postoperative day; rNPT, renal negative pressure treatment.)

During rNPT, 5 patients had 6-hour CrCl measured. With the exception of patient D, CrCl remained unchanged or improved during the 24-hour rNPT treatment (Figure 2A). Patient D was an outlier with baseline SCr concentration of 216 μmol/L and eGFR of 25 mL/min per 1.73 m^2^ (stage 4 CKD). During rNPT, CrCl ranged from 6 to 16 mL/min. Criteria for stage 1 AKI were met with an SCr concentration of 265 μmol/L on POD 3. In the remaining 6 patients, SCr concentration and eGFR were stable or improved at 72 hours and PODs 14 and 28 (Figures 2B, 2C). At 28 days, 4 patients remained in stage 3a CKD, 1 had improved from stage 3a to stage 2, and 1 improved from stage 3b to stage 3a (Supplemental Table 2). Patient D remained in stage 4, albeit with eGFR worsened from 25 to 16 mL/min per 1.73 m^2^.

**Supplemental Figure 2:**
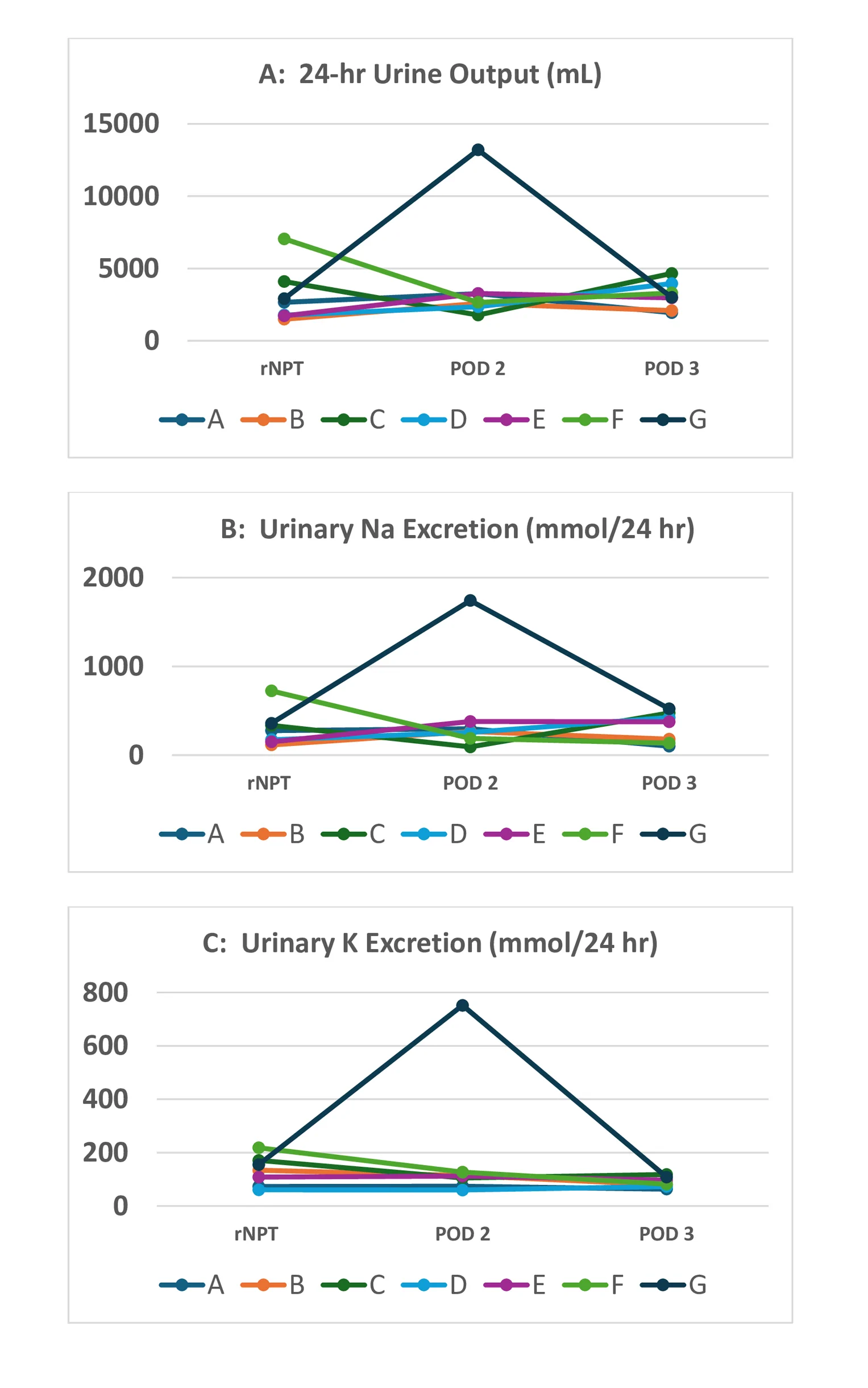
Urine output, sodium and potassium excretion. (A) 24-hr urine output in mL in the efficacy population (patients A-G). (B) Sodium excretion in mmol/24 hr in the efficacy population (patients A-G). (C) Potassium excretion in mmol/24 hr in the efficacy population (patients A-G). 24-hour urinary output was at or above 2L during rNPT in all patients. Patient G was given two 10 mg doses of oral torsemide on POD 2 that evoked a 13L diuresis with concomitantly increased urine sodium and potassium excretion. (POD, postoperative day; rNPT, renal negative pressure treatment).

Urine output and sodium and potassium excretion during rNPT and on PODs 2 and 3 are illustrated in Supplemental Figure 2. The 24-hour urine output was at or above 2 L during rNPT in all patients.

## Comment

Determinants of renal dysfunction after cardiac surgery include preexisting CKD, operative complexity, prolonged CPB, perioperative cardiac dysfunction, and hemodynamic instability.^1^ Current therapy is limited to guidelines, such as optimizing hemodynamic function and avoidance of hyperglycemia, nephrotoxic drugs, and renin-angiotensin system blockade.^1^

Specific interventions to prevent or to reverse renal injury must address its pathophysiologic mechanism. In cardiorenal syndromes, GFR is impaired by inadequate renal arterial perfusion or renal venous congestion.^2^^,^^3^ Aggressive diuretic regimens are commonly used to treat severe CHF, with mixed results.^7^ Investigative devices use intravenous catheters to decrease venous return to the kidneys or to promote aquapheresis.^4^

rNPT provides a more direct approach to enhance GFR. In a porcine study, application of rNPT enhanced diuresis, natriuresis, and measured GFR with or without experimental CHF.^5^ In a subsequent human study, 7 patients with symptomatic CHF and diuretic resistance were treated with rNPT at −15 mm Hg for 24 hours and responded with a significant increase in urine output and sodium excretion.^6^ In a porcine study of sham cardiac surgery with CPB, low urinary oxygen tension was significantly less frequent in animals randomized to rNPT,^8^ postulating a renoprotective effect beyond simple reduction of renal venous pressure.

This study demonstrates that rNPT in patients undergoing cardiac surgery with CPB is safe if there is strict adherence to the catheter placement protocol, which would be further enhanced by inclusion of a periodic catheter position check. Microhematuria was a consistent finding; 2 patients had hematuria that was mild and transient and spontaneously resolved. This might imply that ureteric catheterization is not completely benign; but without controls, we were unable to compare its risk in cardiac surgery with CPB without rNPT.

Despite preexisting CKD, 6 of the 7 per-protocol patients had stable or improved renal function at PODs 14 and 28. AKI developed in 1 patient with stage 4 CKD, an imminent risk in patients with this severity of CKD with or without surgery. Postoperative application of rNPT was unable to reverse the injury but may have prevented the patient from requiring renal replacement therapy.

Limitations to this study include its small size, the lack of a matched untreated control group with which to compare adverse effects and efficacy, and the postoperative application of rNPT. These are being addressed by an ongoing randomized controlled study (NCT07017933) providing rNPT throughout cardiac surgery, potentially revealing a renoprotective effect. It is also important to identify the population most likely to benefit from rNPT, whether related to severity of CKD, CHF, or right-sided heart failure.
