# Supplementary material for: Feasibility Study of Renal Negative Pressure Treatment After Cardiac Surgery With Cardiopulmonary Bypass
Source: Ann Thorac Surg Short Rep. 2026 Feb 26;4(3):1047–52. doi: 10.1016/j.atssr.2026.01.017 (PMC13555481; doi:10.1016/j.atssr.2026.01.017)
Supplement: Supplementary file 1 [file mmc1.docx]

**Supplemental Table 1: Urine Color**

| **Patient** | **Screening** | **Pre-treatment** | **rNPT hrs** | | | | | | **POD** | | | |
| --- | --- | --- | --- | --- | --- | --- | --- | --- | --- | --- | --- | --- |
|  |  |  | **0-8** | | **8-16** | | **16-24** | | **2** | **3** | **14** | **28** |
|  |  |  | **JF** | **BD** | **JF** | **BD** | **JF** | **BD** | **Bladder** | | | |
| ***Protocol Deviation Patients*** | | | | | | | | | | | | |
| 1 | Y | Y | Y | LY | LY | LY | Y | Y | Y | LY | LY | Y |
| 2 | LY | LY | Y | LY | Y | Y | Y | Y | LY | Y | Y | Y |
| 3 | Y | Y | Y | Y | Y | Y | Y | Y | Y | Y | Y | Y |
| ***On Protocol Patients*** | | | | | | | | | | | | |
| A | Y | Y | LY | LY | LY | LY | LY | Y | LY | LY | Y | LY |
| B | LY | LY | Y | LY | Y | LY | LY | * | LY | LY | Y | Y |
| C | LY | Y | Y | Y | Y | Y | Y | Y | Y | Y | LY | Y |
| D | LY | LY | LY | LY | LY | * | LY | * | Y | LY | LY | Y |
| E | LY | Y | LY | LY | Y | * | LY | * | LY | LY | Y | LY |
| F | Y | CY | LP | R | CY | P | LY | P | LY | Y | Y | LY |
| G | LY | LY | LY | Y | P | Y | LY | P | Y | Y | LY | Y |

Urine color (light pink, pink or red) revealed 6 episodes of transient hematuria during rNPT in patients F and G in the safety population. Two episodes occurred from the JuxtaFlow® catheters, four from the bladder collection bag and all resolved within the next 8-hour collection period.

rNPT = renal Negative Pressure Treatment

JF = JuxtaFlow® drainage, BD = bladder drainage

POD = postoperative day

CY = clear yellow, LY = light yellow, Y = yellow, LP = light pink, P = pink, R = red, * = missing

**Supplemental Table 2: eGFR and CKD Stage**

**in Per-Protocol Population**

| **Patient** | **Age** | **Sex** | **SCr Baseline** | **SCr**  **POD 14** | **SCr POD 28** | **eGFR Baseline** | **CKD Stage** | **eGFR POD 14** | **CKD Stage** | **eGFR POD 28** | **CKD Stage** |
| --- | --- | --- | --- | --- | --- | --- | --- | --- | --- | --- | --- |
| A | 68 | F | 150 | 129 | 105 | 31 | 3B | 37 | 3B | 47 | 3A |
| B | 65 | F | 102 | 97 | 91 | 50 | 3A | 53 | 3A | 57 | 3A |
| C | 81 | M | 105 | 114 | 101 | 57 | 3A | 52 | 3A | 60 | 2 |
| D | 74 | M | 216 | 223 | 314 | 25 | 4 | 24 | 4 | 16 | 4 |
| E | 74 | F | 103 | 107 | 94 | 46 | 3A | 44 | 3B | 52 | 3A |
| F | 70 | F | 99 | 103 | 102 | 50 | 3A | 48 | 3A | 48 | 3A |
| G | 69 | M | 116 | 118 | 114 | 55 | 3A | 54 | 3A | 56 | 3A |
