# Supplementary material for: Feasibility Study of Renal Negative Pressure Treatment After Cardiac Surgery With Cardiopulmonary Bypass
Source: Ann Thorac Surg Short Rep. 2026 Feb 26;4(3):1047–52. doi: 10.1016/j.atssr.2026.01.017 (PMC13555481; doi:10.1016/j.atssr.2026.01.017)
Supplement: Supplementary file 2 [file mmc2.docx]

**Supplemental Figure 1: Patient Population**

**Supplemental Figure 2: Urine Output, Sodium and Potassium Excretion**

(**A**) 24-hr urine output in mL in the efficacy population (patients A-G).

(**B**) Sodium excretion in mmol/24hr in the efficacy population (patients A-G).

(**C**) Potassium excretion in mmol/24hr in the efficacy population (patients A-G).

rNPT = renal negative pressure treatment, POD = postoperative day.
